# Increase of Hospital Mortality in Patients On Mechanical Ventilation in the Biennium 2012-2013: An Observational Study

**DOI:** 10.1186/2197-425X-3-S1-A524

**Published:** 2015-10-01

**Authors:** D García Huertas, F Manzano Manzano, MM Jiménez Quintana, C Rodríguez Mejías, F Santiago Ruiz, F Villagrán Ramírez, A Ruiz Perea, M Colmenero Ruiz

**Affiliations:** Hospital Virgen de las Nieves, Granada, Spain; Hospital Universitario San Cecilio, Granada, Spain

## Introduction

A large number of patients in intensive care unit (ICU) require mechanical ventilation (MV) due to various conditions and have a high mortality. to reduce the high mortality, identification of risk factors is important, as well as temporary evolution of mortality.

## Objectives

To evaluate the relationship between two periods of time and hospital mortality in patients requiring MV in an ICU.

## Methods

A prospective, single-center, observational study was undertaken in patients under MV≥ 24 h in two periods (2010-2011 and 2012-2013) in the 26-beds medical-surgical ICU at the Virgen de las Nieves University Hospital of Granada (Spain). Primary outcome measurement was hospital mortality. the main independent variable was the period 2010-2011and 2012-2013. Other variables were APACHE II score, gender, age, surgery, hepatic cirrhosis, neoplasia, cause, duration of MV, and length of stay in ICU. Hazard ratio (HR) of hospital mortality were calculated.

## Result

The study included 845 patients (371 in first period and 474 in second period). Baseline characteristics were similar between groups. Hospital mortality was significantly higher in the second to the first period: 50.4% (239/474) vs 43.7% (162/371) (non-adjusted HR 1.45, 95% CI, 1.17-1.79, p < 0.001). in the multivariate adjusted model the period 2012-2013 in comparison with the period 2010-2011 was a significant independent predictor of hospital mortality (adjusted HR, 1.60; 95% CI, 1.21 to 2.12; p = 0.005). APACHE II score (1.07; 95% CI, 1.07 to 1.09; p < 0.001), age (1.011; 95% CI, 1.001 to 1.022; p = < 0.001), neoplasia (1.47; 95% CI, 1.008 to 2.15; p = 0.045), hepatic cirrhosis (1.78; 95% CI, 1.08 to 2.95; p = 0.02), and surgery admission (0.52; 95% CI, 0.33 to 0.81; p = 0.004), were also in the model.

## Conclusions

In our research, hospital mortality in patients under mechanical ventilation is high. Additionally, an increase in mortality has been identified in the last biennium of unknown cause.

